# Clustering-Constrained ICA for Ballistocardiogram Artifacts Removal in Simultaneous EEG-fMRI

**DOI:** 10.3389/fnins.2018.00059

**Published:** 2018-02-13

**Authors:** Kai Wang, Wenjie Li, Li Dong, Ling Zou, Changming Wang

**Affiliations:** ^1^School of Information Science and Engineering, Changzhou University, Changzhou, China; ^2^Changzhou Key Laboratory of Biomedical Information Technology, Changzhou, China; ^3^Key Laboratory for NeuroInformation of Ministry of Education, School of Life Science and Technology, University of Electronic Science and Technology of China, Chengdu, China; ^4^Beijing Anding Hospital, Beijing Key Laboratory of Mental Disorders, Capital Medical University, Beijing, China

**Keywords:** neuroimaging, EEG-fMRI, ballistocardiogram artifacts, clustering algorithm, constrained ICA

## Abstract

Combination of electroencephalogram (EEG) recording and functional magnetic resonance imaging (fMRI) plays a potential role in neuroimaging due to its high spatial and temporal resolution. However, EEG is easily influenced by ballistocardiogram (BCG) artifacts and may cause false identification of the related EEG features, such as epileptic spikes. There are many related methods to remove them, however, they do not consider the time-varying features of BCG artifacts. In this paper, a novel method using clustering algorithm to catch the BCG artifacts' features and together with the constrained ICA (ccICA) is proposed to remove the BCG artifacts. We first applied this method to the simulated data, which was constructed by adding the BCG artifacts to the EEG signal obtained from the conventional environment. Then, our method was tested to demonstrate the effectiveness during EEG and fMRI experiments on 10 healthy subjects. In simulated data analysis, the value of error in signal amplitude (*Er*) computed by ccICA method was lower than those from other methods including AAS, OBS, and cICA (*p* < 0.005). *In vivo* data analysis, the Improvement of Normalized Power Spectrum (*INPS*) calculated by ccICA method in all electrodes was much higher than AAS, OBS, and cICA methods (*p* < 0.005). We also used other evaluation index (e.g., power analysis) to compare our method with other traditional methods. In conclusion, our novel method successfully and effectively removed BCG artifacts in both simulated and *vivo* EEG data tests, showing the potentials of removing artifacts in EEG-fMRI applications.

## Introduction

Simultaneous recording of electroencephalogram (EEG) and functional magnetic resonance imaging (fMRI) can make full use of the complementarity between the high temporal resolution of EEG and the millimeter spatial resolution of fMRI when studying brain activity (Jorge et al., 2014; Murta et al., 2015). Simultaneous EEG-fMRI acquisition is an important tool for further understanding of brain function and dysfunction including neurofeedback (Zotev et al., 2014), recognition memory, epilepsy (Dong et al., 2015, 2016), and schizophrenia (Ford et al., 2016) etc. However, this neuroimaging technique has one disadvantage that two major artifacts, gradient artifacts (GA) and ballistocardiogram (BCG) artifacts, can be induced on EEG data. GA can be removed by average artifact subtraction (AAS) (Allen et al., 2000). However, BCG artifacts removal is hard due to its non-stationary nature. As mentioned in Allen et al. (1998), Debener et al. (2008), and Mullinger et al. (2013), the mechanisms of BCG artifacts generation are most likely related with the electrodes movement caused by heart beat inside the static magnetic field of the MRI scanner, presenting an evident interpersonal variance character. The amplitude of the BCG artifacts is proportional to the intensity of the magnetic field inside the MRI scanner (Yan et al., 2010; Mullinger et al., 2013) and its shape changes over time (Debener et al., 2008). These characteristics of the BCG artifacts make it hard to predict and characterize, causing troubles in artifact removal. Furthermore, the EEG signals mainly at alpha frequencies (8–13 Hz) and below are easily contaminated by BCG artifacts (Bonmassar et al., 2002), which are important for our study. Therefore, in EEG-fMRI applications, BCG artifact removal is a meaningful issue and more difficult than GA removal (Laufs et al., 2008).

Currently, there are two main approaches to remove the BCG artifacts, namely time domain methods and spatial-pattern techniques. For example, spatial filtering schemes based on a principal component analysis (PCA) and an independent component analysis (ICA) use the statistic signatures of the BCG artifacts to remove artifacts (Bénar et al., 2003; Niazy et al., 2005; Srivastava et al., 2005). Other techniques just use the quasi-periodic of the BCG artifacts, like average artifact subtraction (AAS) (Allen et al., 2000), optimal basis sets (OBS) (Niazy et al., 2005), moving general linear models (mGLMs) (Vincent et al., 2007), and adaptive filtering techniques (Bonmassar et al., 2002). However, spatial-pattern techniques are time-consuming, while waveform removal techniques depend highly on the user experience. In addition to traditional techniques shown above, a reference-removal of BCG artifacts with harmonic regression (Krishnaswamy et al., 2016), the dictionary learning method (Abolghasemi and Ferdowsi, 2015), the real-time OBS method (Wu et al., 2016), and the specific data recording called DRPE (direct recording prior encoding) (Xia et al., 2014) are also widely used to remove BCG artifacts to achieve better separation of BCG and EEG. On the other hand, decreasing the electrode movements can be considered as an important method to reduce BCG artifacts (Allen et al., 1998).

To the best of our knowledge, blind source separation methods can also be used to remove BCG artifacts. The ICA method can decompose EEG signals into independent components (ICs) and subtract the artifacts-related ones to remove the artifacts (Nakamura et al., 2006; Mantini et al., 2007). However, the problems of ICA-based methods are non-reproducibility of the results (Briselli et al., 2006; Grouiller et al., 2007) and the choice of artifacts-related ICs are based on subjective experience. In order to resolve the problem of component selection, Leclercq et al. (2009) developed a new technique based on ICA called constrained ICA (cICA). They set a constrained condition that made the component selection more intelligent and could furthest select the most similar BCG artifact-related components. As mentioned in their article, the method demonstrated more robustness and was computationally more efficient than other ICA-based or traditional methods. But the cICA method didn't put the variability of artifacts into consideration, and there may be residual artifacts.

In this article, we propose a novel method of clustering-constraint ICA (ccICA) to remove the BCG artifacts which takes into account artifacts' time-varied shape, amplitude, and scale. The efficacy of the ccICA was tested in simulated and real data and compared with traditional OBS, AAS and cICA methods. The results for both time and frequency domains showed that our method is promising in removing BCG artifacts from EEG data recorded in MRI environment.

## Materials and methods

### Data acquisition and subjects

In order to verify the effectiveness of our method, acquisition of EEG-fMRI data from 10 healthy subjects (age range: 20–25; 7 males and 3 females) was applied. In all recordings, the subjects lay relaxed in the scanner with eyes closed and head fixed. The subjects were given written notice and consented to the participation in this study. The experiments were carried out in accordance with the Declaration of Helsinki (2000) of the World Medical Association and the protocols approved by the Institutional Review Board at Changzhou University. Data acquisition was done with EEG amplifier and MR scanner offered by Changzhou Key Laboratory of Biomedical Information Technology.

We also collected three additional task-EEG data to verify the efficiency of the method by extracting ERP “N170” from them. “N170” is a “face-sensitive” event-related potential (ERP) that occurs at around 170 ms over occipito-temporal brain regions. The experiment included 56 trials and the duration of each trial was 4 s. The subjects were first asked to look at some face images in learning stage, and then some other face images were shown in the screen. The subjects chose “1” if they saw the face before; otherwise, they chose “2.”

Functional images were obtained from the subjects on a 3.0T scanner (Magnetom Sonata, Philips, Holland) with parameters: TR = 2000 ms, TE = 35 ms, Flip angle = 90°, FOV = 230 × 180 mm^2^. Each volume consisted of 24 slices. EEG data were acquired using an MR compatible EEG amplifier (EGI, America) with sampling rate of 1,000 Hz and a cap with 64 Ag/AgCl electrodes positioned according to the system provided by EGI. In addition, an extra electrode was placed in the chest for electrocardiogram (ECG) signal acquisition as well. All channels were online referenced to Cz. These data were collected during continuous fMRI using an echo planar imaging (EPI) sequence.

### Ballistocardiogram (BCG) artifacts

BCG artifacts are caused by cardiac-related activities which distort the EEG signals in the static magnetic field. Its causes and characteristics are described in literature (Allen et al., 1998; Bonmassar et al., 2002). In general, the electrodes' movement is caused by heart beat-related activities (Allen et al., 1998) and the magnitude of the BCG artifacts may be as much as 400 μV in 3.0T (6–8 times that of EEG) (Allen et al., 2000). Moreover, considerable variation of BCG artifacts' shape, amplitude, and scale over time was another characteristic observed.

### BCG removal algorithm

In this section, our novel method as well as reported cICA was applied to remove the BCG artifacts. Since every technology needs to detect the QRS peak in ECG, the same QRS detection algorithm for each of them is used, and details are described in the next section. QRS peak detection, AAS and OBS algorithm implementation throughout FMRIB plug-in were provided by the University of Oxford Centre for Functional MRI of the Brain. Available techniques used for comparison are shown below before introducing our algorithm.

#### QRS peak detection

The earliest ECG R-peaks detection algorithm was put forward by Allen et al. (1998) in simple thresholding detections. However, they were defective due to time-consumption and being poorly robust in the MR environment. Thus, Christov (2004) proposed a combined adaptive threshold algorithm for the detection of the ECG R-peak. Niazy et al. (2005) modified this algorithm by computing a complex lead from some ECG channels. The ECG channel should be band-pass filtered from 7 to 40 Hz firstly, and then the complex lead was calculated by using a *k*-Teager energy operator (*k*-TEO) (Mukhopadhyay and Ray, 1998; Kim et al., 2004) to the filtered ECG channel and by setting all negative values to zero, (see Equation 1):

(1) $$\begin{array}{r} ECG\left(n\right)=\max\left(E^{2}\left(n\right)-E\left(n-k\right)E\left(n+k\right),0\right) \end{array}$$

Where *ECG* is the complex lead, *n* is the time index, *E* is the filtered ECG, and *k* is a frequency selection parameter (Kim et al., 2004). Next, the combined adaptive thresholding algorithm was applied (Christov, 2004) to detect the peaks. The sum of the steep-slope threshold (*M*), the integrating threshold for high-frequency signal components (*F*), and the beat expectation threshold (*R*) are used as the *MFR* threshold, (see Equation 2):

(2) $$\begin{array}{r} MFR=M+F+R \end{array}$$

When *ECG(n)*≥*MFR(n)*, it will be detected as a QRS peak. For more information about the QRS peak detection, please check the original paper (Niazy et al., 2005).

#### Clustering combined with cICA (ccICA)

BCG artifacts could be identified and removed using classical ICA algorithms; however, component selection in ICA is easily influenced by subjective factors and sources have to be estimated through cumbersome iterative search. In contrast, newly proposed cICA can be used to identify BCG-related sources through finding the closest source to a given constraint (Leclercq et al., 2009). This indicates that the identified components closely match the constraint and thus this consumes less computational cost. Moreover, the whole process was marked by its automated, data driven and not observer-dependent nature.

The constraint set in cICA algorithm assumes that BCG artifact is a quasi-periodic signal, which is less practical and does not fully capture the characteristics of BCG artifacts. In cICA framework, for each channel an average BCG-artifact template is calculated across successive EEG signal by a simple average, regardless of the beat-to-beat variability in heart rate and shape and amplitude differences of BCG artifacts.

**Figure 1:**
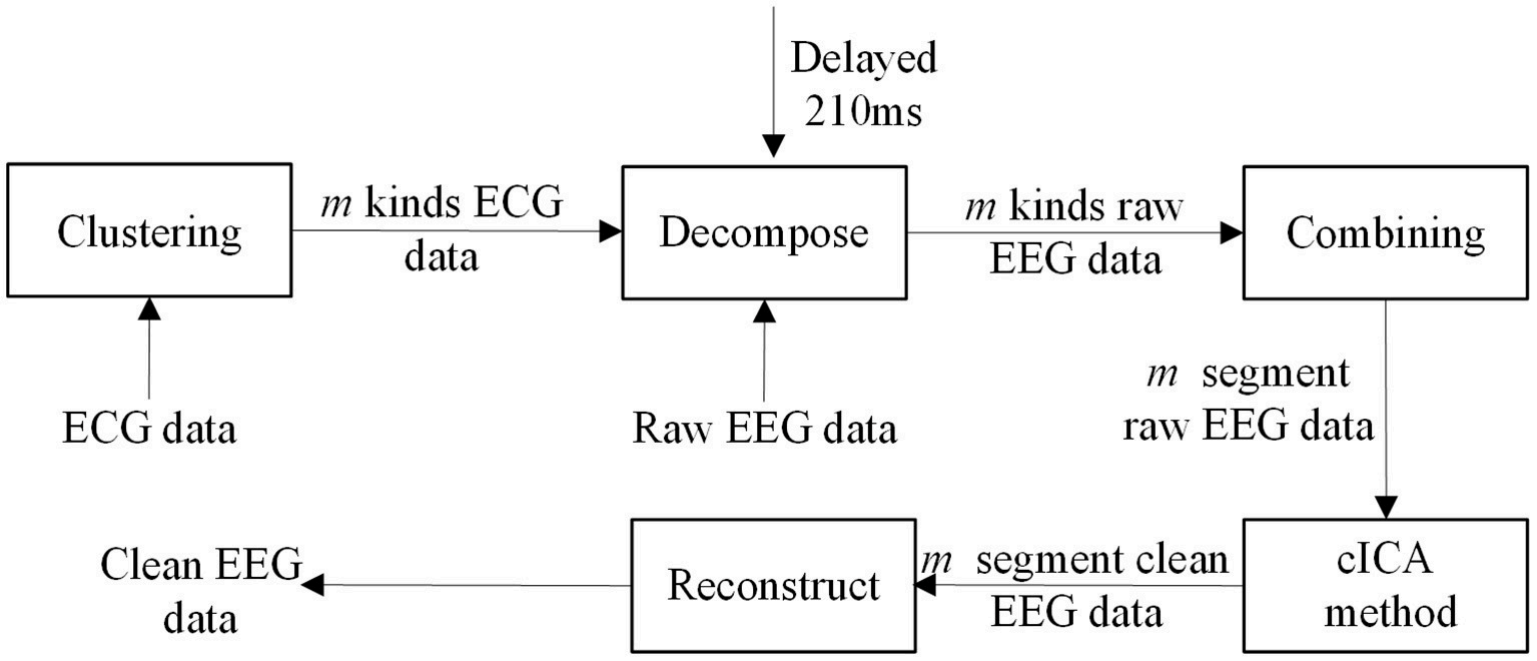
The block diagram of ccICA method.

Hence, in this study a clustering-constrained ICA (ccICA) method is proposed. In ccICA, clustering algorithm is applied to classify the BCG artifacts into different types and to detect outliers before cICA. In ccICA, signals from ECG channel are first segmented according to Wu et al. (2016). Then, the hierarchical clustering (Ward, 1963) algorithm is used to subdivide the ECG segments into *L* groups. The distance between every pair of ECG segments (can be measured by either Single Linkage or Average Linkage) is computed and a distance table is obtained. It is then used to determine the optimal separation of a partition of ***L***, satisfying that data variability within each cluster is minimized. The clustering algorithm then assign each ECG segment to a specific cluster. Given the relationship between BCG artifacts in EEG signals and ECG, the positions of BCG artifacts in EEG signal are determined referring to the positions of R-peak in ECG, i.e., if the R-peak's location is *Ri* (in time), the corresponding BCG artifacts in EEG is identified in *Ri* + 210 ms (Allen et al., 1998). In this way, the BCG artifacts are extracted and separated into ***L*** groups referring to ECG segments. Finally, by combining the clustering results, the constrained ICA (cICA) algorithm (Leclercq et al., 2009) is used to get corrected EEG signals. Figure 1 shows a simple block diagram of our ccICA method. In Figure 1, the ECG data will be classified into *m* kinds using clustering algorithm, then the raw EEG data will also be classified into *m* kinds due to the relationship which has been described above. The raw EEG epoches in one cluster are combined continuously. Hence, the raw EEG data will be recombined into *m* segments. Then, the cICA algorithm will be applied into each raw EEG segment. Last, all clean EEG segments are decomposed into epoches according to the clustering labels to reconstruct the corrected EEG data.

According to cICA Leclercq et al. (2009), cICA assumes that the EEG signal is a linear mixture of statistically independent artifacts sources and neural sources. The number of sources should be equal to the number of channels, and their size is defined to be [*N* channels × *T* time points]. **D** is the recorded EEG signals [*N* channels × *T* time points], and **W** is a fixed scalar matrix. The goal of the ICA is to find the **W**, which is a square matrix of size *N*.

The row vectors of **W** can be viewed as a non-orthonormal base of an *N*-dimensional space. During preprocessing, an orthonormal matrix $\tilde{W}$ is deducted from **W**. The identification of the artifact-related row-vectors in $\tilde{W}$ would make it possible to find the complementary orthonormal set of vectors. More details of the cICA algorithm could refer to Leclercq et al. (2009).

### Evaluation index

To evaluate the efficacy of the clustering combined with cICA method, several evaluation analyses were conducted, including time domain and frequency domain. Some evaluation indexes are described below.

#### Error in signal amplitude

The error in signal amplitude is an important technical index to evaluate the efficacy between our method and other methods. The error in signal amplitude (*Er*) after BCG artifacts removal is defined as:

(3) $$\begin{array}{r} Er=\frac{1}{n}\overset{n}{\underset{i=1}{\sum }}|x\left(i\right)-y\left(i\right)| \end{array}$$

where *x(i)* is the amplitude of sample *i* in the data after BCG artifacts removal, and *y(i)* is the amplitude of *i* in the original EEG data. Moreover, this equation could be used to compare different methods.

#### Sensitivity *(Se)* and specificity *(Sp)*

For the ECG recording, the occurrence of actual ECG R-peak was determined through a visual inspection. The total count of QRS complexes was considered as the true positive number, *T*_*P*_. The QRS complex which was not detected by the algorithm was considered as a false negative, *F*_*N*_. Another condition detected in the absence of an actual QRS complex was considered as a false positive, *F*_*P*_. If the position of the detected QRS complex had a time-shift error, it was also considered as false negative or false positive. The *Se* and *Sp* were used to demonstrate the accuracy of the ECG detection algorithm and the quality of our data. Both were calculated for each data-set as follows:

(4) $$\begin{array}{r} Se=\frac{Tp}{Tp+F_{N}} \end{array}$$

(5) $$\begin{array}{r} Sp=\frac{Tp}{Tp+Fp} \end{array}$$

#### Power spectra

To compare the efficiency of the new method with the AAS, OBS, and cICA methods, the power spectra of the signals before and after algorithm applied was evaluated. Meanwhile, the obtained signals after applying different methods were also compared. The power spectrum was calculated as

(6) $$\begin{array}{r} S\left({\text{e}}^{jw}\right)=\frac{1}{n}{\left|\overset{n}{\underset{l=1}{\sum }}x_{l}e^{-jwl}\right|}^{2} \end{array}$$

where e^*jw*^ represents a superposition of a cosine signal with a sinusoidal signal. *S*(e^*jw*^) is the power spectrum, and x_*l*_ is the value of sample *l* in each EEG channel.

#### Improvement of normalized power spectrum *(INPS)*

Another quality index for assessing BCG artifacts removal algorithms quality is the Improvement of Normalized Power Spectrum(*INSP*)introduced by Tong et al. (2001), which indicates the power attenuation in the corrected signal:

(7) $$\begin{array}{r} INPS\left(Chan,N\right)=\frac{\overset{N}{\underset{j=1}{\sum }}P_{j}^{before}}{\overset{N}{\underset{i=1}{\sum }}P_{i}^{after}} \end{array}$$

where *P*^*before*^ and *P*^*after*^ are mean power in a 1 Hz window centered on the *j*th harmonic of the heart frequency respectively before and after BCG artifact removal for a particular channel *Chan*. The *INPS* indicates the power attenuation, the larger *INPS* means that more power has been reduced. However, the larger *INPS* may not indicate reduction of more artifacts, and the useful signals may also be reduced.

#### Relative root mean square error (*RRMSE*)

Relative root mean square error is also calculated in each method and compared quantitatively. The RRMSE is defined as follows (Li et al., 2013; Despotovic et al., 2016):

(8) $$\begin{array}{r} RRMSE=\frac{\sqrt{\frac{1}{n}\overset{n}{\underset{i=1}{\sum }}{\left({\bar{H}}_{d}^{i,m}-{\bar{H}}_{d}^{i,c}\right)}^{2}}}{\overset{n}{\underset{i=1}{\sum }}{\bar{H}}_{d}^{i,m}}\times 100 \end{array}$$

Where ${\bar{H}}_{d}^{i,c}$ is the *ith* calculated value, which represents the corrected signal. ${\bar{H}}_{d}^{i,m}$ is the *ith* measured value, which is the raw signal. *n* is the sample points.

## Results

### Simulated data

Simulated data consisted of original EEG signal (obtained out of the MRI scanner) and varying BCG artifacts. Moreover, the original data (without fMRI) without adding the BCG artifacts were compared. In general, the construction of the simulated data can be divided into three steps.

First, we averaged all the artifact segments from one channel of real EEG data without removing the BCG to generate the BCG artifact template. Second, we randomly and independently scaled the BCG template in terms of amplitude and time span to simulate the varying BCG artifacts. Finally, varying BCG artifacts were added to the original EEG data (without fMRI) to generate the simulated data. The interval between the peaks and their amplitude were randomized.

The *Er* values we calculated according to different methods are shown in Table 1.

**Table 1:** The *Er* of BCG artifact removal using different methods.

**Method**	***Er* (μv)**
OBS(*pc* = 4)	19.32
AAS	19.10
cICA	9.38
ccICA	8.45

In Table 1, it can be seen that the *Er* of the method we proposed is much lower than other methods. Both the AAS method and OBS method have much higher *Er* values than ours. Further comparison between cICA and ccICA is needed.

**Figure 2:**
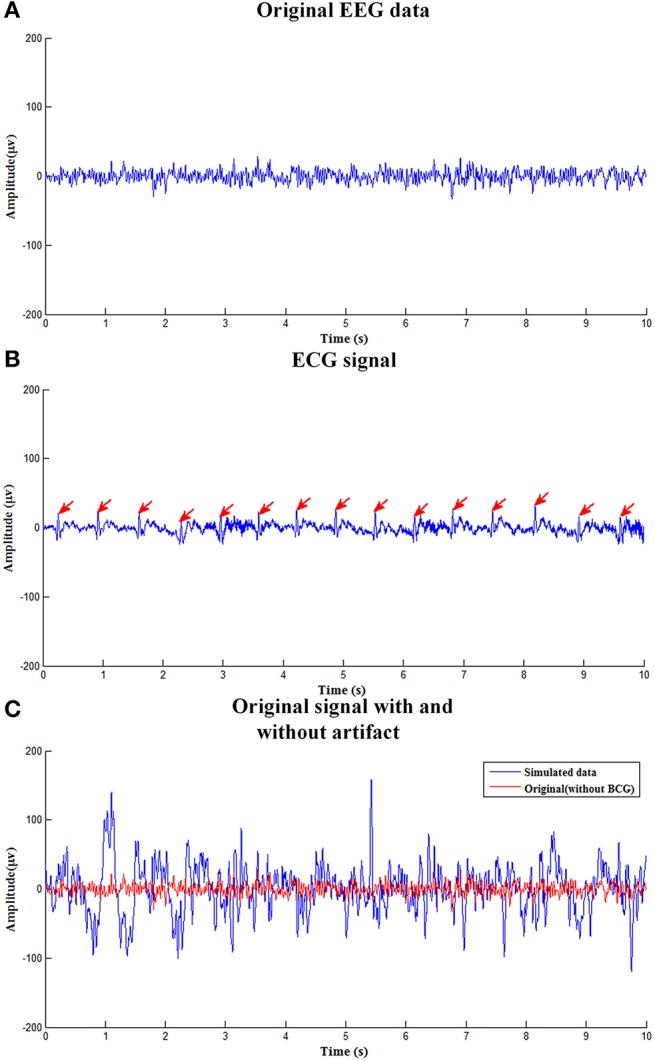
The simulated EEG signal. **(A)** The clean EEG data used to construct the simulated contaminated EEG data. **(B)** The ECG signal. **(C)** Original signal with and without BCG artifact.

Figure 2 shows simulated EEG signals with and without adding BCG artifacts as well as corresponding simulated ECG signal. Figure 2A shows the original single channel (O1 electrode) EEG data used to construct the simulated data obtained in the isolated room with 50Hz-frequency interference filtered. Figure 2B is the simulated ECG signal detecting the R-peak, as marked with the red arrows. Figure 2C shows the simulated EEG signal with BCG artifacts used to evaluate the efficiency of the ccICA method.

**Figure 3:**
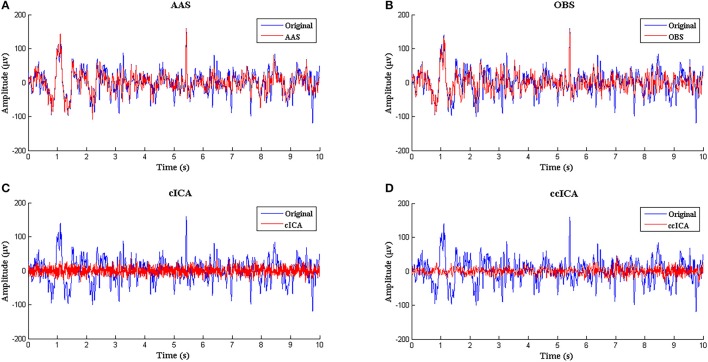
Performance comparison between ccICA and other methods. **(A)** BCG artifact removal using AAS (red line). **(B)** BCG artifact removal using OBS (*pc* = 4) (red line). **(C)** BCG artifact removal using cICA (red line). **(D)** BCG artifact removal using ccICA (red line).

Figure 3 shows the simulated signal before and after BCG artifact removal using four different methods respectively. The blue curves denote the simulated signals without artifact removal and the red curves denote the signals after BCG artifacts removal. From the vision of Figures 3A,B, the residual artifact is very obvious and some BCG artifacts can't be removed. Figures 3C,D show the results using cICA and ccICA, both methods can remove almost all BCG artifacts, but compared with Figure 2C, the signal tendency after using ccICA method is more uniform. In other words, the signal after using ccICA closely matches the original EEG signal. Hence, the method we proposed seems to provide cleaner signals after BCG artifacts removal.

### In vivo

All real data were collected simultaneously using fMRI, including 64 channels EEG data and one channel ECG data. The simultaneous ECG data were used to detect the BCG artifacts' locations in EEG data. The gradient artifacts were removed by AAS (Allen et al., 2000). The sensitivity (*Se*) and specificity (*Sp*) are index to illustrate the robustness of R-peak detection algorithm proposed by Niazy et al. (2005).

Table 2 illustrates the sensitivity and specificity of the ECG R-peak detection algorithm. The first seven data's average *Se* and *Sp* are more than 99%, suggesting the robustness of R-peak detection algorithm (Christov, 2004) amended by Niazy et al. (2005) in detecting ECG R-peak in the analysis. The data of subject eight had a visible difference due to the fall of ECG electrodes or some other reasons. Therefore, this kind of data was abandoned.

**Table 2:** The sensitivity (*Se*) and specificity (*Sp*) of the R-peak detection algorithm.

**Subject No**.	***Se* (%)**	***Sp* (%)**
1	99.61	99.80
2	99.57	99.79
3	99.43	100
4	99.05	99.80
5	99.50	99.75
6	100	99.78
7	99.74	100
8	86.78	83.96
Avg	97.96	97.86
Std	4.53	5.62
Std(without subject 8)	0.29	0.11

#### BCG artifact removal using ccICA method

**Figure 4:**
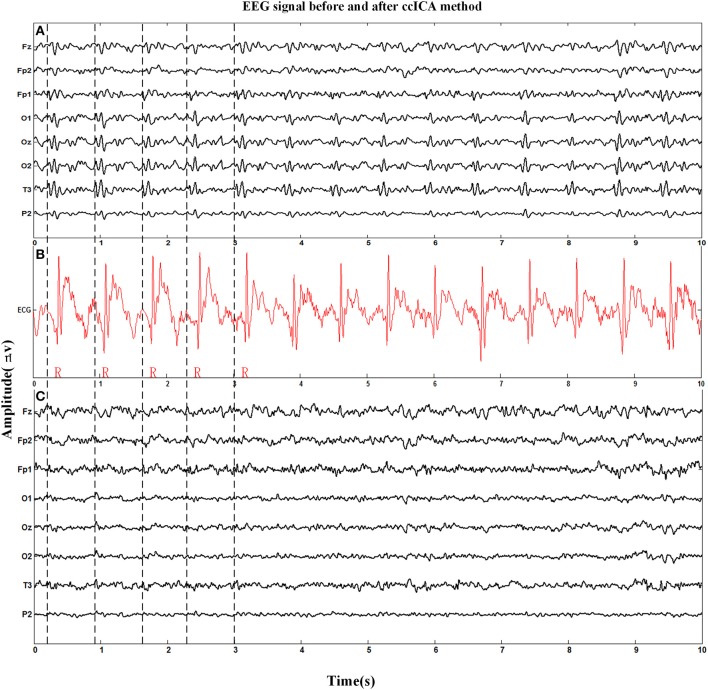
Eight channel EEG signals before and after BCG artifact removal applied by ccICA method and one channel ECG signal is used to mark the location of R-peak and BCG artifact. **(A)** Signals before BCG artifact removal. **(B)** The ECG signal. **(C)** Signals after BCG artifact removal by ccICA method.

Figure 4 shows the eight channel signals (Fz, Fp2, Fp1, O1, Oz, O2, T3, P2, the electrode location was placed according to the EGI system, similar with 10/10 system, only some electrodes with different name) obtained from an MRI scanner while the subjects were requested to keep a resting state with their eyes closed. Figure 4A is the signal before applying the ccICA method, Figure 4B is the corresponding ECG signal marked with red line, and Figure 4C is the signal after BCG artifact removal. A part of the locations of the R-peak are tabbed by the vertical dashed lines. It can be seen that BCG artifacts appear after the recent R-peak from Figures 4A,B (about 210 ms according to Allen et al., 2000). But in Figure 4C, BCG artifacts are absent according to visual observation.

**Figure 5:**
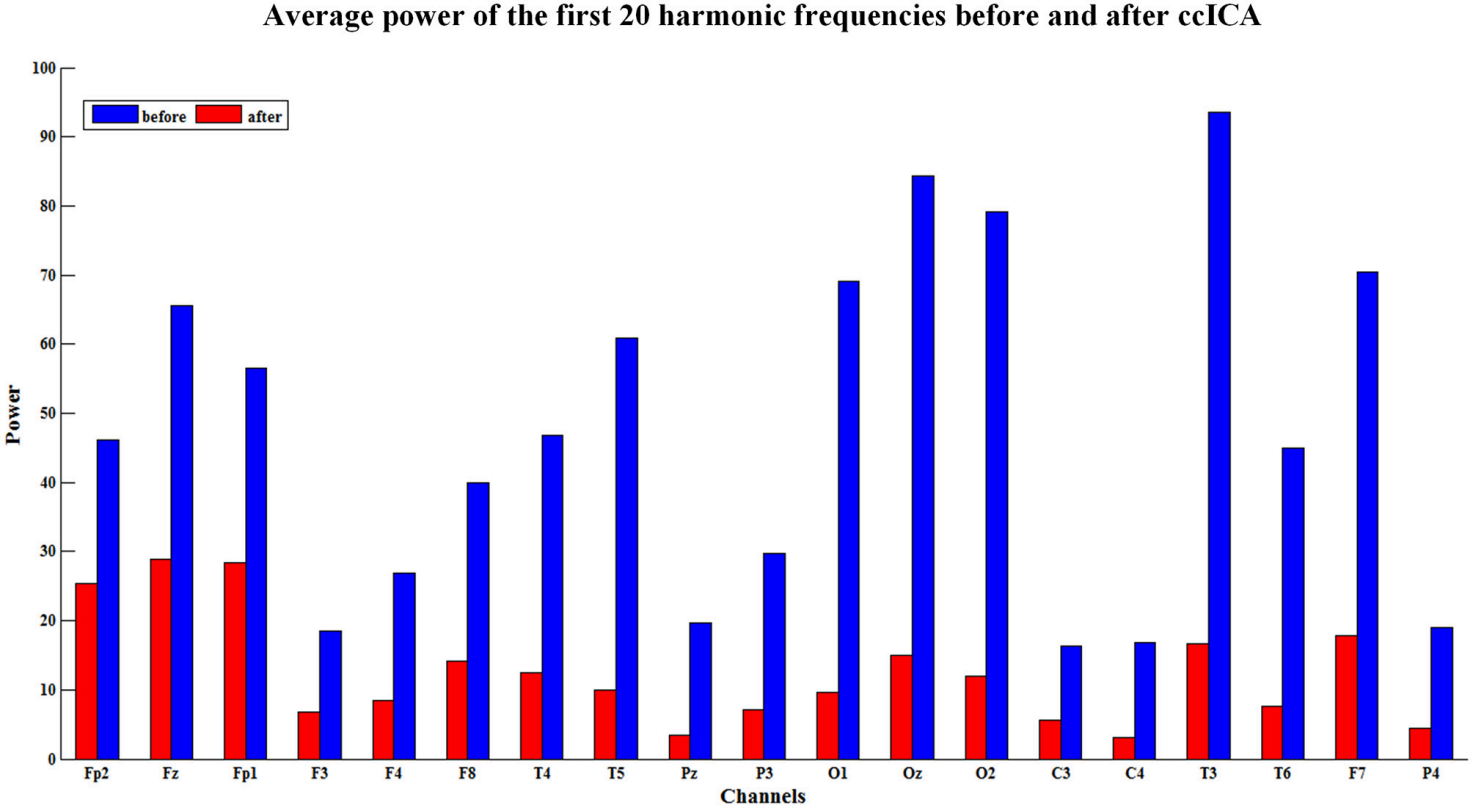
The average power of the first twenty harmonic frequencies before (blue bars) and after (red bars) using ccICA method, it indicates that the BCG artifacts have been suppressed in a large part.

The spectral power is also an important index to evaluate the efficiency of the BCG artifacts removal method. The spectral power in EEG harmonic frequencies was calculated for each electrode and each subject before and after BCG artifacts removal using ccICA. Nineteen electrodes signals were examined on 10 subjects and the average spectral power for first 20 harmonic frequencies of the 10 subjects were computed. Figure 5 shows the results of the average power spectral before and after the ccICA method employed. The histograms indicate that the BCG fundamental (~1 Hz) and harmonic powers decrease significantly. In other words, the contamination of BCG artifacts has been effectively suppressed in a large part.

#### Comparisons between ccICA, cICA, OBS, and AAS methods

**Figure 6:**
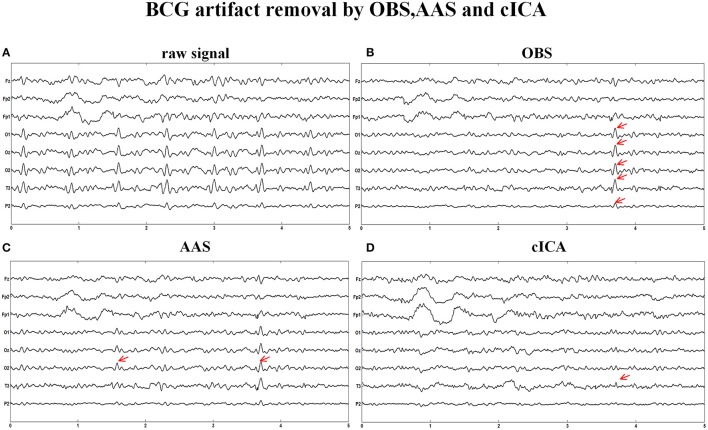
BCG artifacts removal using other methods. **(A)** Signals without removing BCG artifacts. **(B)** BCG artifacts removal using OBS and the part of residual artifacts were marked with red arrows. **(C)** BCG artifacts removal using AAS **(D)** BCG artifacts removal using cICA.

Figure 6 illustrates the same signals handled by other methods. In Figure 6A, the raw signals are not treated with BCG artifacts removal algorithm, and regular artifacts are noticeable. Figures 6B,C show the signals after OBS and AAS method, residual BCG artifacts and some parts are marked with the red arrows. Combining the results of the simulated signal, we can draw a conclusion that the AAS and OBS method do not live up to the ideal result. The cICA method can remove most BCG artifacts, but persevere some residual artifacts, which are marked with red arrows.

**Figure 7:**
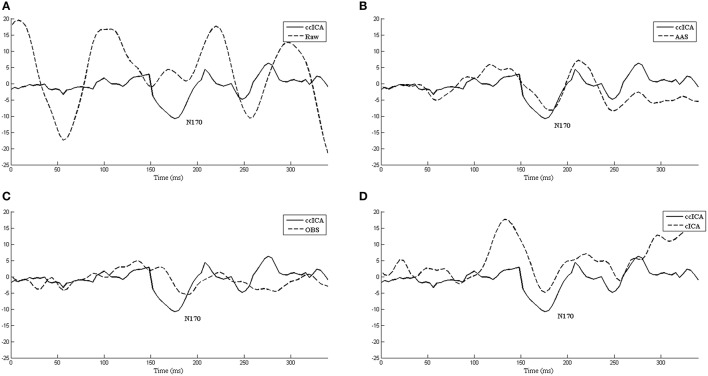
The ERP “N170” extracted by different methods and comparisons between them. **(A)** Raw signal and ccICA method. **(B)** AAS method and ccICA method. **(C)** OBS method and ccICA method. **(D)** cICA method and ccICA method.

We also extract the event-related-potential (ERP) “N170” from the EEG signal after being corrected by AAS, OBS and cICA methods. The results are shown in Figure 7. Figure 7A shows the comparison between raw signals and signals corrected by the ccICA method. The signal is significantly contaminated by the artifacts. In Figure 7B, “N170” extracted by the AAS method is blurry, an analogous negative wave appears in 250 ms. Therefore, the result may be ambiguous. In Figure 7C, the OBS method can also extract “N170.” However, “N170” appears later. The “N170” extracted by cICA method is shown in Figure 7D, however, a large sine wave appears before 150 ms, which may be influenced by artifacts. Moreover, we computed the signal-to-noise ratio (SNR) (Shams et al., 2015) of “N170,” and the results are shown in Table 3. From the results described above, we can conclude that the ccICA method can reduce large BCG artifacts and preserve more EEG signals. However, due to the complex environment in MRI scanner, our method seems unable to completely remove the artifacts, and we can also see the oscillation near “N170.” So, the results may be different from the conditions in the conventional environment (Sadeh and Yovel, 2010).

**Table 3:** The SNR of “N170” computed by different methods.

**Method**	**AAS**	**OBS**	**cICA**	**ccICA**
SNR(*dB*)	1.46	0.79	1.54	3.21

**Figure 8:**
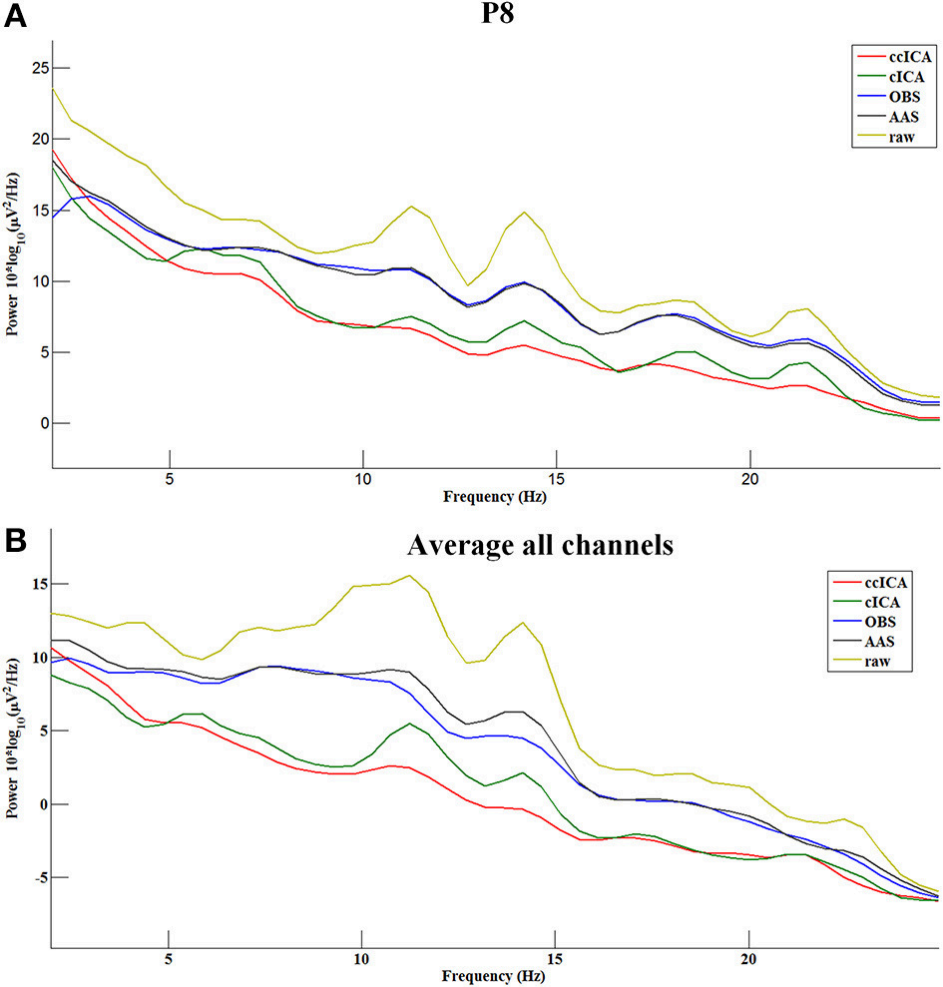
Power spectrum of the signals before and after using all methods. **(A)** Electrode P8. **(B)** Average all channels.

In the frequency domain, the signal power has a significant attenuation after being applied with every method (Figure 8). Figure 8A shows the P8 electrode signal's spectral power before and after using all methods and Figure 8B shows the average spectral power of all channels. From Figure 8, ccICA (red line) and cICA (green line) methods remove more power in all frequency bands than OBS (blue line) and AAS (black line) methods. Moreover, ccICA removal efficiency is better than cCIA in α and β frequency band.

**Figure 9:**
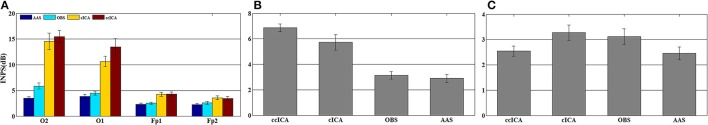
Mean INPS over the 10 subjects. **(A)** Mean INPS over the 10 subjects of different electrodes from 8 to 12 Hz. **(B)** Mean INPS over the 10 subjects of all electrodes from 8 to 12 Hz. **(C)** Mean INPS over the 10 subjects of all electrodes from 1 to 5 Hz.

**Figure 10:**
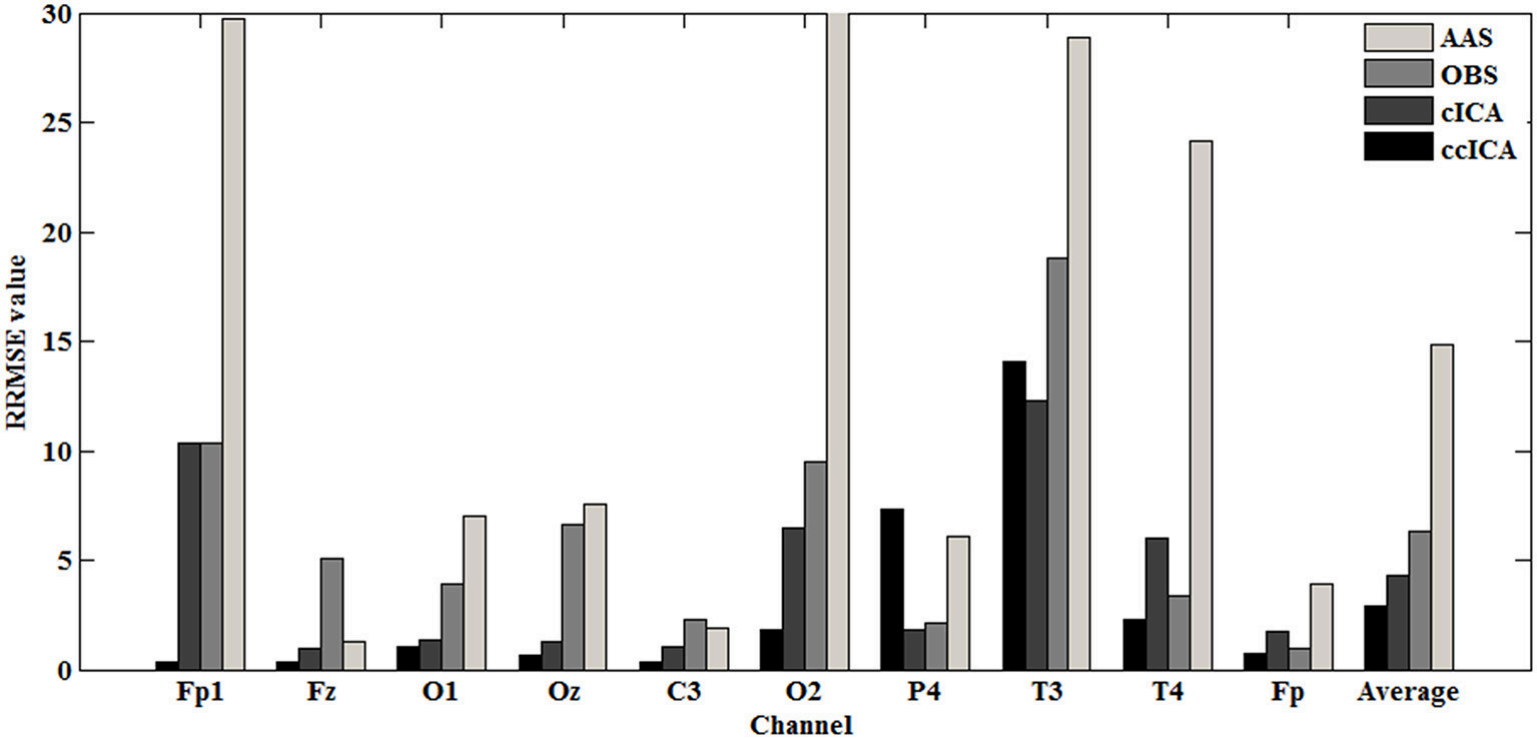
*RRMSE* value in each channel.

**Figure 11:**
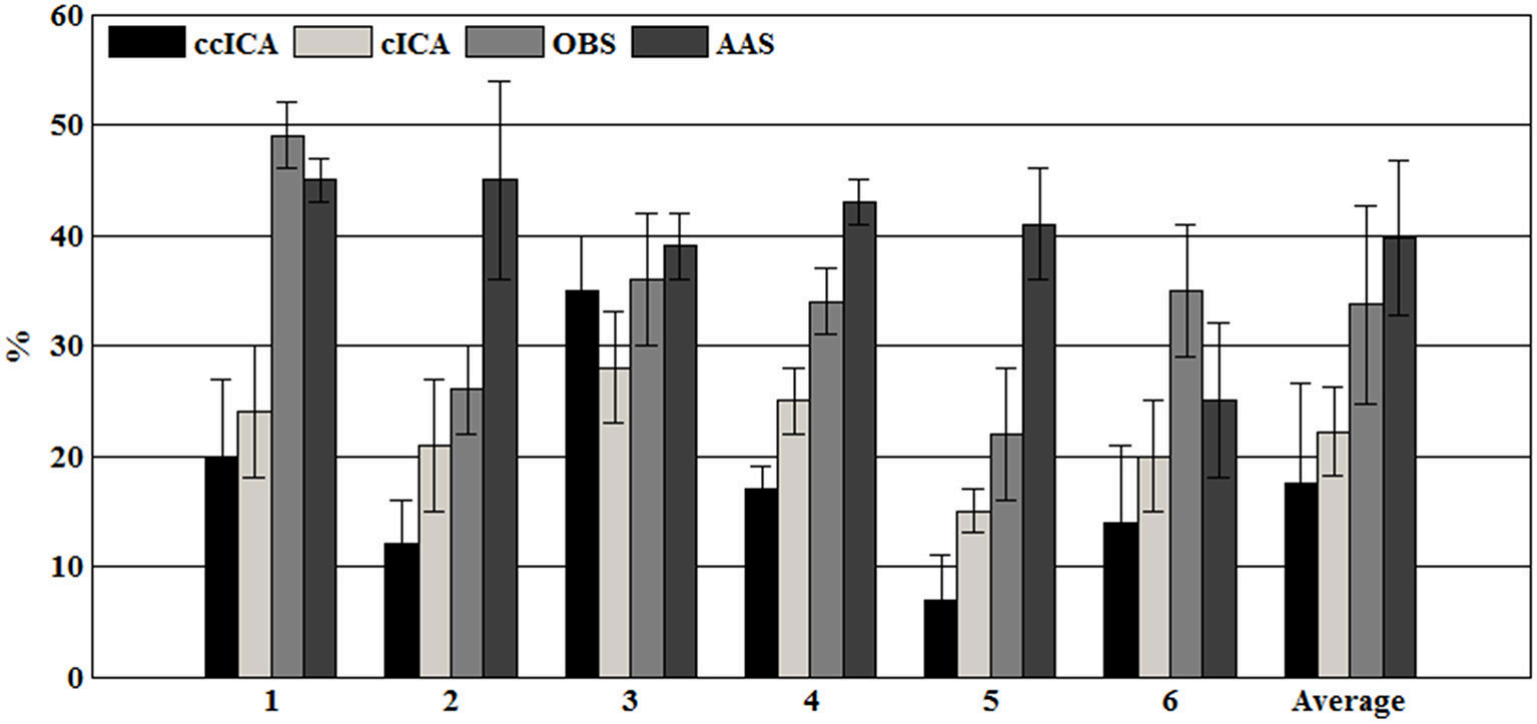
The residual BCG artifacts power ratio in each subject.

For computing the *INPS*, we choose the *N* as 5 because in literature *N* is usually chosen between 4 and 6 (Srivastava et al., 2005; Briselli et al., 2006; Nakamura et al., 2006). Figure 9 shows the result of *INPS*. All these are statistically significant (*p* < 0.005). Figures 9A,B show the mean *INPS* for the four methods across four electrodes signals and all electrodes of all subjects from 8 to 12 Hz, indicating that the ccICA method rejects more power than other methods (expect cICA in Fp2), especially in Occipital region. From Figure 9B, power attenuation in low frequency region (~from 1 to 2 Hz) after applying ccICA is lower than cICA or OBS but higher than AAS (OBS non selectively suppresses a large amount of power around 1 Hz, suggesting cICA method may perform much better in low frequency band). As shown in Figure 9, the ccICA method corrected signals are significantly different from the raw signals in every frequency region and every tested electrode. Also, we compute the *RRMSE* value by using simulated data. From the result of Figure 10, the averaged RRMSE value obtained by ccICA is less than 10%, it indicates that the method is excellent. Also, we could conclude that ccICA performs better than other methods. Another index can clearly compare the efficiency of different methods. Figure 11 summarizes the residual BCG artifacts power ratio from the four methods in six subjects (Niazy et al., 2005). The result shows that the residual BCG artifacts after applying the ccICA method is obviously lower than other methods. It indicates that ccCIA method remove more BCG artifacts. It is desirable to study the method which performs better in the frequency band than other methods in future. For more evaluation index, please refer to (Shams et al., 2015; Krishnaswamy et al., 2016) and related articles.

To further verify the impact of the clusters number and kind of clustering algorithm on our result, we compute the *Er* value use different number of clusters and kind of clustering algorithm, the result is shown in Table 4. The result show that three clusters is suitable for our data and k-means algorithm seems more robust for classifying BCG artifacts.

**Table 4:** The *Er* value of BCG artifacts removal using different clustering methods and the number of clusters.

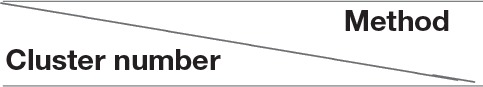	**Hierarchical clustering**	**k-means**
2	61.41	55.75
3	28.68	40.67
4	73.65	75.41
5	92.38	52.61
6	72.21	60.54

## Discussion

Simultaneous EEG and fMRI is a powerful technique in functional neuroimaging. It is widely used in examining brain activity and numerous studies. Due to the benefits of this technique, a series of challenges have arisen. In particular, practical implementations of methods for the removal of the main obstacles GA artifacts and BCG artifacts are highly worthwhile. However, the current removal algorithm AAS and OBS have many defects. AAS may remove useful signals and preserve many residual artifacts. Moreover, OBS method depends on subjective selection, which also affects the EEG signal quality.

In this paper, considering the huge variations of BCG artifacts, we propose a novel method based on constraint ICA. Although ccICA is based on the classic ICA machinery, the efficiency has outperformed the ICA algorithm. The main advantages of ccICA are: (1) take the BCG artifacts variability into consideration; (2) component selection is not interfered by subjective factors, which improve the extraction accuracy of artifact-related components; (3) the method is more efficient and robust in BCG artifacts removal and outperforms traditional methods in many aspects. As mentioned in Leclercq et al. (2009), this method has one disadvantage that when the EEG channel is less, the result will be uncorrected.

### R-peak detection

Robust R-peak detection algorithm is crucial in BCG artifacts removal. In general, the R-peak detection algorithms can be classified into three main categories: threshold algorithm (Christov, 2004; Niazy et al., 2005), matching algorithm and syntactical algorithm. Considering the variations of BCG artifacts, the combined adaptive threshold algorithm (Christov, 2004) amended by Niazy et al. (2005) can determine the R-peaks accurately. The algorithm can consider the variations in the intervals between adjacent ECG peaks, while another two methods are not applicable. Additionally, as shown in Table 2, the *Se* and *Sp* of ECG R-peak detection we calculated in the real data are more than 99% (expect damaged ECG data, i.e., Subject 8 in Table 2). In addition, the ECG segment based on the detected R-peaks and accurate ECG segments can make the clustering algorithm more efficient.

### Compare with cICA, OBS and AAS

Performance comparisons between the ccICA method and cICA, OBS, AAS (Allen et al., 2000; Niazy et al., 2005; Leclercq et al., 2009) were based on same simulated data and real data, guaranteeing effective comparability of the results shown in section Results. According to the results, significant changes have taken place in signals before and after BCG artifacts removal when using ccICA method, and the results are much better. It indicates that the EEG signal obtained in the MRI environment after applying the ccICA method is much cleaner. Consistently, comparing the results of ccICA and other methods in time domain or frequency domain, we find that the ccICA method can effectively suppress the BCG artifact-related components and preserve the useful EEG neuro-related components, such as alpha rhythm and beta rhythm.

In our novel BCG artifacts removal method, we catch the variations of BCG artifacts and classify them into different categories using clustering algorithm, which has been neglected by other methods. Therefore, our method can provide more accurate estimates of the BCG artifacts components and improve the EEG signals quality after BCG artifacts removal, whereas the fixed BCG artifact template used by other methods is insufficient for estimating BCG artifact components.

Clustering algorithm is used to classify BCG artifacts, but the number of the clusters to be chosen is worth considering. Different numbers may lead to different results. Moreover, the use of different clustering algorithm will lead to different BCG artifacts classifications.

Therefore, it is necessary to find the optimal clustering algorithm and the suitable number of clusters. Also, due to the complex environment in the MR scanner, ccICA can't completely remove the artifacts. From Figure 7, we can see the significant oscillation near “N170” caused by residual artifacts, the residual artifacts' amplitude and scale being very similar to the EEG signal. This kind of components may be artifacts or EEG signal when using ICA algorithm. If ccICA identifies this kind of components as the artifacts, it will remove some useful EEG signals. Hence, more details should be considered.

For the blind source separation used to remove BCG artifacts, many other strategies are proposed for artifacts removal. For example, an optical motion-tracking system was used to measure the BCG motion (LeVan et al., 2013) or a mark-based method to correct BCG artifacts (Körbl et al., 2016). Also, a method based on BCG reference layer (BRL) and standard EEG cap was applied (Luo et al., 2014; Krishnaswamy et al., 2016). Some real-time methods also performed well in BCG artifact removal (Mayeli et al., 2016; Wu et al., 2016). It is worthwhile to note that during the removal of artifacts, the physiological signal preservation should be taken into account (Abreu et al., 2016). Therefore, the way to suppress the generation of artifacts by hardware is recommended. In our study, we improved the efficiency of well-studied ICA based method. Our method showed improvements of BCG artifact removal in simulated or real data. In future studies, the demonstration of validity of our method is worthwhile.

### Limitations

The current study shows that our ccICA method can efficiently remove BCG artifacts and does better than traditional methods. However, it also has some potential limitations, such as the cluster types, the type of clustering algorithm (only use hierarchical clustering and k-means in this article) we should choose, and different choices may affect the results. In addition, different reference of scalp EEG recordings, such as REST reference (Yao, 2001) and Cz reference, is also a factor that may be taken into consideration. Meanwhile, sampling rate and R-peak detection algorithm also result in a different addition. For our method, a little distortion of corrected EEG data may happen when we reconstruct the corrected EEG data. When extracting the ERP of “N170,” we also met some problems so that the “N170” had a little distortion. It may be caused by the low SNR of the EEG signal or some useful EEG signals were removed. Meanwhile, the other unknown artifacts also affect the quality of “N170.” Low sampling rate is worth considering whether it affects the results. Therefore, BCG artifacts removal is more challenging because many factors should be taken into consideration.

## Conclusions

In this paper, a novel ICA-based technique is introduced for removing the BCG artifacts. The clustering algorithm is adopted, making the BCG artifacts-related selections more effective. The results demonstrate the effectiveness of ccICA in both the time domain and the frequency domain. Comparisons between ccICA and other methods also suggest that our method can remove BCG artifacts more effectively and obtain a much cleaner EEG signal.

## Author contributions

KW: Organized and wrote the manuscript; WL: Developed and implemented the computational methods and conducted the experiments; KW and WL: discussed the main idea and contributed equally to it; LD: Gave helpful suggestions for improving the paper; LZ and CW: Supervised all aspects of the work; All authors read and approved the final manuscript.

### Conflict of interest statement

The authors declare that the research was conducted in the absence of any commercial or financial relationships that could be construed as a potential conflict of interest.
